# A 30 m Multi-Year Dataset of Major Crop Distributions in Xinjiang, China (2013–2024) Based on Harmonized Landsat–Sentinel-2 Data

**DOI:** 10.1038/s41597-026-07082-w

**Published:** 2026-03-23

**Authors:** Qixiang Liang, Yanfeng Di, Xingming Hao, Jingjing Zhang, Mengtao Ci, Fun Sun, Chuan Wang, Xue Fan, Xinran Guo

**Affiliations:** 1 State Key Laboratory of Desert and Oasis Ecology, Key Laboratory of Ecological Safety and Sustainable Development in Arid Lands, Xinjiang Institute of Ecology and Geography, Chinese Academy of Sciences, Urumqi, 830011 China; 2 University of Chinese Academy of Sciences, Beijing, China; 3 Aksu National Station of Observation and Research for Oasis Agro-ecosystem, Aksu, Xinjiang China; 4 School of Geography and Ocean Science, Nanjing University, Nanjing, 210023 China; 5 Key Laboratory of Carbon Neutrality and Territory Optimization, Ministry of Natural Resources, Nanjing, 210023 China

## Abstract

Accurate and timely information on the spatial distribution of crops is essential for ensuring food security, achieving sustainable agricultural management, and understanding ecosystem interactions. However, in large-scale arid regions like Xinjiang, China, constructing high-spatial-resolution, continuous, and multi-year crop distribution datasets remains a significant challenge due to complex terrain, sparse ground observations, and limited computational resources. In this study, we developed a robust crop classification framework leveraging the Google Earth Engine (GEE) cloud platform. The framework integrates all available NASA–Sentinel-2 (HLSL30) imagery to construct harmonic models based on NDVI and LSWI indices, effectively characterizing crop phenological trajectories. These features are combined with a Random Forest (RF) algorithm to achieve detailed identification of major crop types. To minimize interference from non-crop vegetation and background land cover, we implemented a pre-extracted cropland mask. Using this approach, we generated a 30 m resolution dataset of major crops (including cotton, maize, wheat, and rice) across Xinjiang for the period 2013–2024. Accuracy assessments using independent validation samples from 2018 and 2019 yielded producer accuracies of 0.83–0.99 and user accuracies of 0.83–0.96. The overall accuracy reached 0.90 and 0.93, with Kappa coefficients of 0.86 and 0.89, respectively. Furthermore, the estimated crop areas at the prefecture level show high consistency with official statistical yearbooks and align well with existing distribution maps of cotton, maize, and wheat. This dataset provides a systematic characterization of the long-term spatial dynamics of major crops in Xinjiang, offering critical and reliable data support for regional agricultural monitoring, food security assessment, policy formulation, and environmental change research.

## Background & Summary

Agriculture plays a central role in linking all Sustainable Development Goals (SDGs), as each of the 17 United Nations SDGs is directly or indirectly connected to agricultural activities^1^. As a major grain production base in western China, Xinjiang possesses approximately 107.87 million mu of arable land—accounting for 5.59% of the nation’s total and 41.74% of that in the five northwestern provinces (Xinjiang, Gansu, Ningxia, Qinghai, and Shaanxi) (Third National Land Survey of China). The region’s dominant crops—cotton, maize, and wheat—collectively occupy about 74.87% of its total cultivated area (2022 Xinjiang Statistical Yearbook). High-precision and up-to-date crop distribution maps are therefore critical for crop yield simulation, food security assessment, and sustainable agricultural decision-making^2^ and for deepening our understanding of crop–ecosystem interactions^3^. However, previous research has primarily focused on mapping single crop types or specific years^2,4–6^, with limited efforts to achieve systematic and continuous multi-crop monitoring across large regions. Developing comprehensive, long-term, and spatially consistent crop distribution maps is thus essential for understanding agricultural dynamics in Xinjiang.

Remote sensing technology, with its advantages of wide spatial coverage, temporal continuity, and cost efficiency, has become the principal tool for regional-scale crop identification and monitoring^7^. Globally, several large-scale crop mapping initiatives have demonstrated its effectiveness, including the Cropland Data Layer (CDL) developed by the U.S. Department of Agriculture^8^, the European Crop Type Map derived from Sentinel-1 data and LUCAS field observations^9^, and the 10-meter crop map of Northeast China for 2017–2019^10^. Extensive remote sensing research on crop identification and mapping has been conducted in Xinjiang, with a predominant focus on cotton distribution mapping. For instance, previous studies have produced a 10 m resolution cotton map for Northern Xinjiang in 2019^11^, a Xinjiang-wide cotton dataset for 2018–2021^2^, and cotton maps for several specific oasis regions within Xinjiang^12^. Furthermore, some research has expanded to multi-crop mapping within typical oasis areas of the region^13,14^. On a broader scale, several national-level crop distribution products covering Xinjiang have been developed. These include a 10 m resolution maize map of China for 2017–2021^15^, a maize planting distribution dataset for China covering 2001–2024^7^, and a 500 m resolution dataset of China’ s three major food crops (maize, rice, and wheat) for 2015–2021^16^. However, data products specifically targeting the major crop distributions across the entire Xinjiang region at a 30 m spatial resolution remain remarkably scarce.

Xinjiang covers an area of approximately 1.6 million km², representing one-sixth of China’s total land area. Its distinct “mountain–basin” topography and fragile “mountain–oasis–desert” ecosystem^17^ create additional complexity for agricultural remote sensing. Furthermore, challenges such as limited ground samples, model transferability, and computational resource constraints^18–20^ have hindered the development of high-precision, region-wide crop mapping. To overcome these limitations, this study proposes an integrated crop classification framework. First, regional cultivated lands were extracted to reduce interference from non-crop vegetation. Second, NASA Harmonized Landsat Sentinel-2 (HLSL30) v2.0 imagery is interpolated and smoothed to reconstruct complete time-series data; finally, harmonic functions are employed to fit crop growth curves, which, combined with a Random Forest (RF) model, enable the precise identification of crop types.

Leveraging the Google Earth Engine (GEE) platform and all available HLSL30 imagery, we generated a 30 m resolution distribution map of major crops in Xinjiang from 2013 to 2024, with ongoing updates maintained. This dataset will provide essential spatial data support for regional crop management, agricultural policy-making, and research into sustainable development.

## Methods

### Study area

**Fig. 1:**
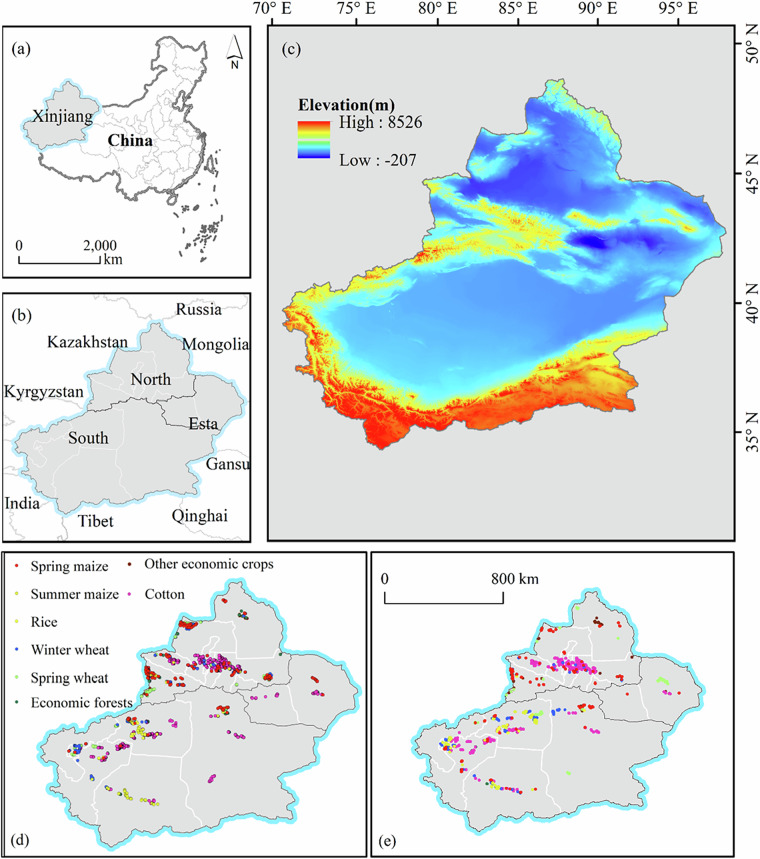
Overview of the study area. (**a**) Geographical location of Xinjiang within China; (**b**) geographical subdivisions and prefecture-level administrative divisions of Xinjiang; (**c**) topographical conditions of the study area; (**d,e**) distribution of crop validation samples in 2018 and 2019.

The study area, Xinjiang Uygur Autonomous Region (73°46′–96°23′E; 34°25′–49°50′N), is located in northwestern China. Covering approximately 1.6 million square kilometers—about one-sixth of the country’s total land area—Xinjiang is the largest provincial-level administrative region in China (Fig. 1a). The region’s terrain is characterized by three major mountain ranges—the Altai Mountains, Tianshan Mountains, and Kunlun Mountains—separated by two vast basins, the Junggar Basin in the north and the Tarim Basin in the south. Administratively, Xinjiang consists of 14 prefectures, autonomous prefectures, and prefecture-level cities (Fig. 1b). Agricultural activities are mainly concentrated in oasis regions, with key irrigation zones distributed across the Ili River Valley, Tarim River Basin, Turpan Basin, and Manas River Basin. Oasis irrigation farming dominates the region’s agricultural production, featuring two primary cropping systems: single cropping and multiple cropping per year. The main cultivated crops include staple grains such as wheat, maize, rice, and soybeans, as well as economic crop such as cotton, oilseeds, sugar beets, vegetables, melons, potatoes, alfalfa, grapes, apples, pears, and jujubes. In recent years, supported by national policies and the adoption of advanced water-saving irrigation technologies, Xinjiang has become China’s largest cotton-producing region^21,22^. In this study, the entire Xinjiang region was selected as the research area. This study utilizes HLSL30 imagery to construct annual crop distribution maps at a 30 m resolution. This provides robust data support for analyzing the spatio-temporal distribution patterns and change characteristics of various crops at a regional scale in Xinjiang.

### Data collection

**Fig. 2:**
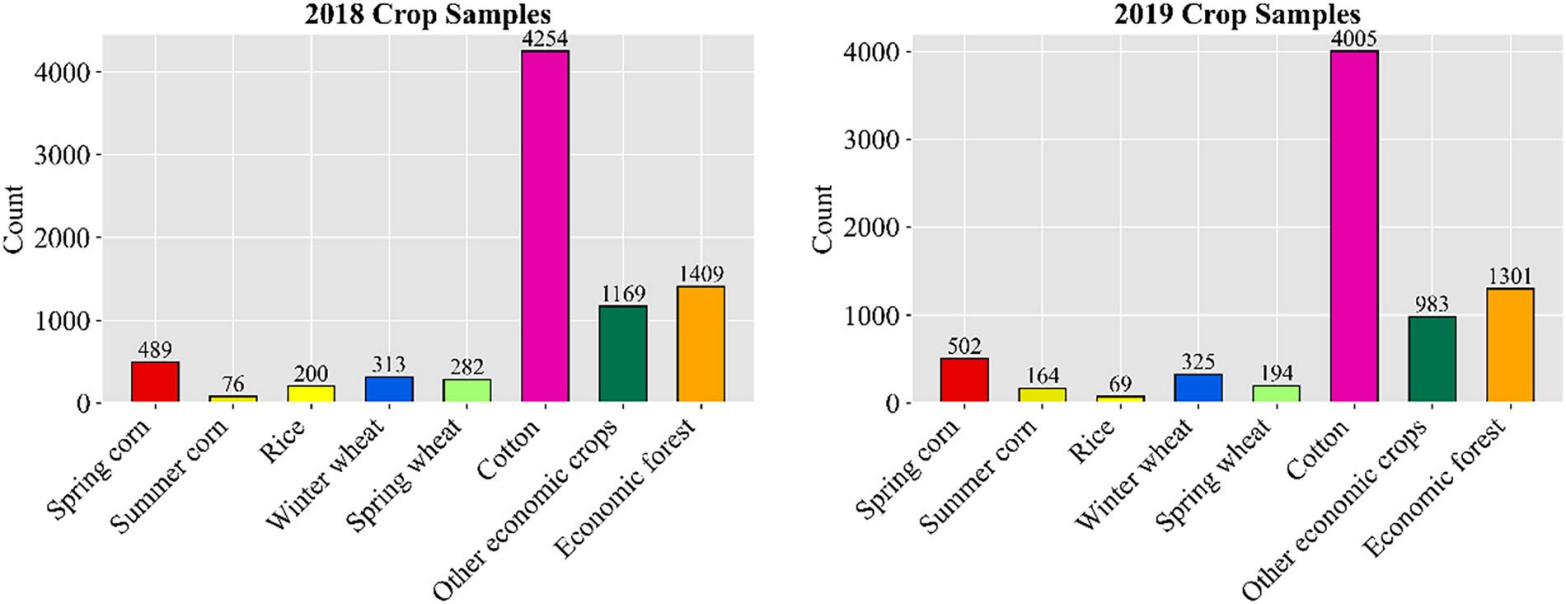
Crop sample counts for 2018 (**a**) and 2019 (**b**).

The crop sample dataset used in this study was collected in 2018 and 2019 through a combination of field surveys and high-resolution remote sensing image interpretation. The samples represent Xinjiang’s major crop types, including spring maize, summer maize, rice, winter wheat, spring wheat, cotton, economic crops, and economic forests EF (Fig. 2a). In 2018, a total of 8,192 samples were collected, corresponding to 489, 76, 200, 313, 282, 4,254, 1,169, and 1,409 samples for each crop type, respectively. In 2019, 7,543 samples were obtained, with 502, 164, 69, 325, 194, 4,005, 983, and 1,301 samples per crop type, respectively (Fig. 2b). The samples were extensively distributed across Xinjiang’s major oasis agricultural regions, encompassing diverse cropping systems and environmental conditions, thereby capturing the spatial variability of the region’s agroecological landscapes and crop diversity. To ensure data reliability and consistency, strict quality control procedures were applied during dataset construction. Samples exhibiting significant geolocation errors, mixed land-cover types, or low-quality imagery were removed. Crop labels were reviewed and corrected through cross-validation and expert-assisted interpretation. In addition, spatial consistency tests and sample balancing procedures were conducted to mitigate potential sampling bias caused by uneven sample size or distribution between years. The resulting dataset demonstrates high reliability in both spatial–temporal representativeness and categorical accuracy. It serves as a reliable reference for subsequent crop mapping and accuracy assessment using HLSL30 data.

The arable land data used in this study were derived from the China 30-meter Annual Land Cover Dataset (CLCD)^23,24^. Produced on the GEE platform, CLCD provides annual land cover information for China from 1990 to 2019 at a 30-meter spatial resolution. The dataset was constructed by integrating stable training samples from China’s Multi-Period Land Use Datasets (CLUDs) with additional samples extracted from satellite time series, Google Earth, and high-resolution image interpretation. More than 330,000 Landsat images were used to extract spectral, phenological, and topographic features, which were then classified using a RF algorithm. To ensure spatiotemporal consistency, a post-processing step combining spatiotemporal filtering and logical inference was implemented to correct anomalous land-use transitions. Validation with 5,463 independent samples yielded an overall classification accuracy of 79.31%. Compared with global land cover products such as MCD12Q1, ESACCI_LC, FROM_GLC, and GlobeLand30, CLCD has demonstrated superior performance, particularly in identifying cultivated lands. Consequently, CLCD provides reliable and temporally consistent information on cultivated land distribution for this study.

Elevation data were obtained from NASADEM^25^, a refined reprocessing of the Shuttle Radar Topography Mission (SRTM) dataset. Its accuracy has been enhanced through the integration of auxiliary datasets, including ASTER GDEM, ICE Sat GLAS, and PRISM.

The HLSL30 product used in this study is a remote sensing dataset jointly launched by NASA and the USGS^26^. It aims to synergistically process data from the Landsat-8 and Sentinel-2 satellites to generate globally consistent surface reflectance data^27^. By fusing the observational capabilities of both satellites, HLSL30 enables global land cover monitoring with a revisit cycle of 2–3 days at a 30 m spatial resolution, making it highly suitable for high-temporal-resolution land change monitoring and analysis^28^. This dataset has been widely applied across various domains, including phenological monitoring^29^, forest and grassland disturbance detection^30,31^, cropland dynamics monitoring^32,33^ and crop mapping^34,35^.

Prefecture-level crop area statistics from the Xinjiang Statistical Yearbook (http://tjj.xinjiang.gov.cn/) were used for area-based validation of the classification results. The yearbook consists of two independent parts: the *Xinjiang Uygur Autonomous Region Statistical Yearbook* and the *Xinjiang Production and Construction Corps Statistical Yearbook*, compiled by local governments and the Corps, respectively. Xinjiang comprises 14 prefecture-level administrative units and 14 Corps divisions. Statistical records spanning 2013–2023 were used in this study for validation and comparison.

The cotton dataset provides 30 m resolution annual cotton distribution maps across major cotton-producing regions of China, generated using multi-source satellite imagery and phenology-based classification methods^2^. The dataset was validated using ground survey samples and high-resolution imagery, achieving high overall classification accuracy and demonstrating strong consistency with statistical records at regional scales. The winter wheat dataset provides 30 m resolution annual maps across 11 major producing provinces from 2001 to 2023, generated using a time-weighted dynamic time warping (TWDTW) method based on multi-source satellite imagery^36–38^. Validation using field and Google Earth samples reported an overall accuracy of 91.17%. The China Crop Distribution – Maize dataset (CCD-Maize) provides annual maize maps from 2001 to 2024 across 22 provinces at 30 m spatial resolution (WGS84, EPSG:4326)^7,39^. The dataset was produced using a TWDTW algorithm applied to fused NDVI time series, with different satellite combinations used for 2001–2020, 2021–2023, and 2024. Based on 54,281 samples, the average overall classification accuracy was 80.06%, and the county-level coefficient of determination (R^2^) between mapped and statistical maize areas ranged from 0.657 to 0.903 (2001–2020).

### Map production process

**Fig. 3:**
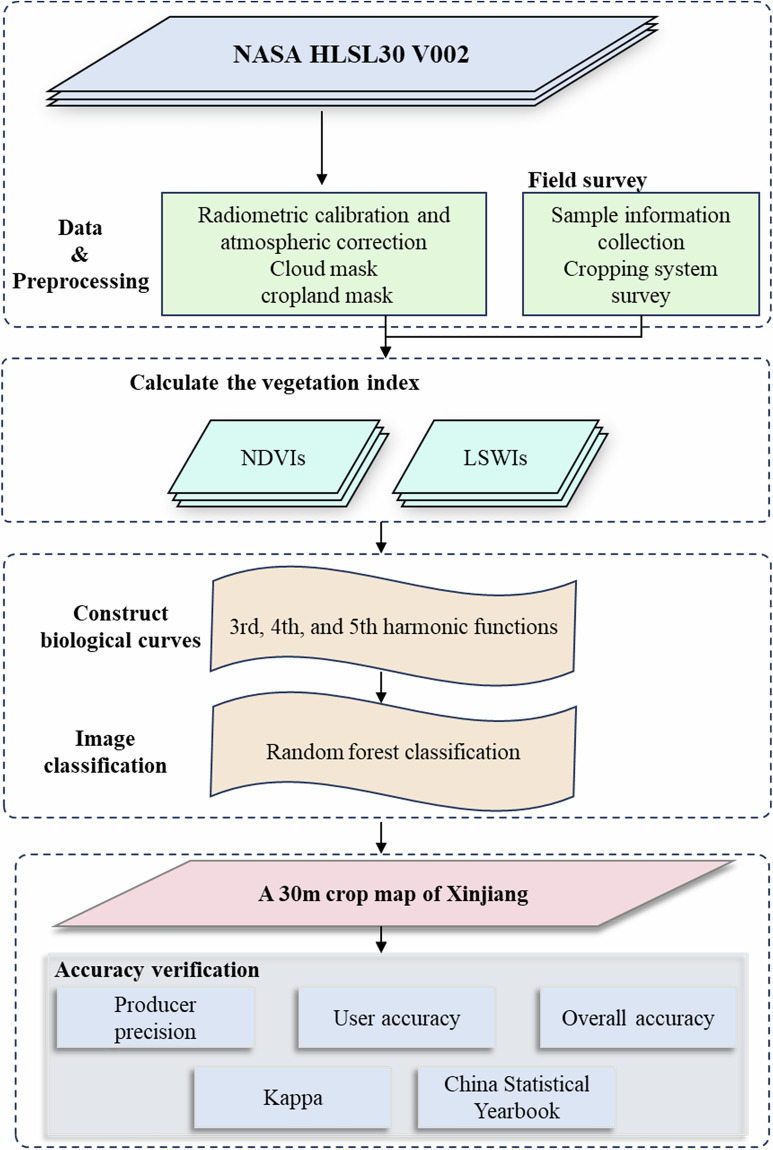
Workflow of the crop mapping framework developed in this study.

Figure 3 illustrates the methodological framework developed in this study for mapping major crops in Xinjiang. The workflow consists of four primary stages:(1) Data Preprocessing: Utilizing the GEE platform, we acquired HLSL30 data products. Based on the built-in Quality Assessment (QA) bands, pixels affected by clouds, cloud shadows, snow, and high aerosol loads were masked. These data were then integrated with a cropland mask to extract potential cultivation areas, resulting in a high-quality, continuous remote sensing time-series dataset for the crop growing season. (2) Sample Construction and Feature Engineering: Crop sample information was compiled from field surveys, historical research, and authoritative statistical records. We analyzed the phenological patterns of major crops in alignment with the specific regional cropping systems of Xinjiang. Subsequently, time-series indices, including the Normalized Difference Vegetation Index (NDVI) and the Land Surface Water Index (LSWI), were calculated. Harmonic analysis was then employed to extract key phenological parameters representing the dynamic growth processes, thereby constructing typical biophenological curves for different crop types in the feature space. (3) Classification and Mapping: The extracted NDVI and LSWI harmonic features, along with the sample data, were input into a RF classifier for training and prediction. This process generated 30 m resolution crop type distribution maps across Xinjiang. (4) Accuracy Validation: The accuracy and reliability of the mapping results were comprehensively evaluated using independent validation samples. Metrics including Overall Accuracy (OA), User’ s Accuracy (UA), Producer’ s Accuracy (PA), and the Kappa coefficient were calculated. Additionally, the classification results were compared and analyzed against official data from the China Statistical Yearbook.

### Crop classification scheme and class definitions

In this study, eight major crop and agricultural vegetation types widely distributed in Xinjiang were mapped, including spring maize, summer maize, rice, spring wheat, winter wheat, cotton, other economic crops (OEC), and economic forests (EF). These classes represent the dominant cropping systems and perennial agricultural vegetation in the region and were defined based on local agronomic practices, planting calendars, and phenological characteristics observable from multi-temporal remote sensing data.

Spring maize and summer maize were treated as separate classes due to their distinct planting schedules and phenological trajectories. Summer maize is typically planted after the harvest of winter wheat, resulting in a delayed green-up and NDVI peak compared to spring maize. Similarly, spring wheat and winter wheat were distinguished based on their different growing cycles and overwintering characteristics.

The category “other economic crops” mainly includes annual or perennial cash crops such as legumes, tuber crops, rapeseed, sugar beet, vegetables, and alfalfa, which exhibit diverse species composition but relatively similar seasonal phenological patterns at the regional scale. The category “economic forests” primarily consists of perennial woody crops and plantations, including jujube, walnut, apple, pear, apricot, and poplar timber forests. These two categories were defined as functional phenology-based classes rather than species-level categories, aiming to enhance classification consistency across large spatial extents. Detailed class definitions are provided in Table 1.

**Table 1:** Definition of crop classes used in this study.

Class name	Included crop/vegetation types	Functional description
Spring maize	Spring-sown maize	Annual cereal crop planted in spring and harvested in late summer; characterized by an early NDVI increase and a single summer peak.
Summer maize	Summer-sown maize	Annual maize planted after winter wheat harvest; exhibits delayed green-up and NDVI peak relative to maize.
Rice	Paddy rice	Flooded annual crop with high NDVI and LSWI values during the growing season.
Spring wheat	Spring wheat	Annual cereal crop with a short and early growing cycle; NDVI peaks in early summer.
Winter wheat	Winter wheat	Overwintering cereal crop with a bimodal NDVI temporal pattern across the growing season.
Cotton	Cotton	Annual economic crop with a long growing season and a pronounced summer NDVI peak.
Other economic crops	Legumes, tuber crops, rapeseed, sugar beet, vegetables, alfalfa, etc.	Heterogeneous economic crops grouped based on similar seasonal phenological characteristics at the regional scale.
Economic forests	Jujube, walnut, apple, pear, apricot, poplar timber forests, etc.	Perennial woody crops and plantations with relatively stable NDVI throughout the year and limited seasonal variability.

### Remote sensing data preprocessing

To generate a high-quality, long-term crop classification dataset, this study utilized HLSL30 data as the primary remote sensing source, processed via the GEE platform. The HLSL30 products integrate Landsat-8/9 OLI and Sentinel-2 MSI data into a consistent 30 m spatial resolution and Military Grid Reference System (MGRS) tiling framework through standardized atmospheric correction, geometric registration, BRDF normalization, and spectral bandpass adjustment. This integration effectively creates a continuous surface reflectance time series from a “virtual single sensor,” significantly enhancing the consistency and comparability of multi-source temporal data^28^. Regarding data quality control, the HLSL30 products include pixel-level Quality Assessment (QA) bands generated using the LaSRC atmospheric correction and the Fmask 4.2 algorithm. The QA band utilizes bit-packed flags to identify clouds, cloud shadows, snow, water bodies, and aerosol optical thickness levels. Following the HLSL30 User Guide, we performed a unified masking process on pixels identified as clouds (bit 1), cloud shadows (bit 3), cloud/shadow buffers (bit 2), snow (bit 4), and moderate-to-high aerosol loads (bits 6–7) to minimize interference from atmospheric and cloud contamination on the temporal signals.After quality masking, to construct a complete and continuous vegetation index time series, linear interpolation was applied to the time series of each pixel under valid observation conditions to fill gaps caused by cloud cover and quality control^40,41^. This preprocessing helps mitigate the impact of high-frequency noise on phenological signals, ensuring the stability and reliability of the subsequent harmonic analysis and phenological feature extraction.

### Feature selection

In crop classification research, vegetation indices serve as vital temporal indicators, effectively reflecting crop growth conditions and water dynamics^29,42^. Drawing on previous studies and crop growth mechanisms, this study selected the Normalized Difference Vegetation Index (NDVI) and the Land Surface Water Index (LSWI) as the primary characteristic variables. Specifically, NDVI characterizes vegetation greenness and photosynthetic intensity, capturing the temporal dynamics of crop growth. In contrast, LSWI is more sensitive to changes in canopy and soil moisture, effectively representing crop responses to irrigation management and moisture stress, thereby compensating for the limitations of NDVI in characterizing water-related information^43,44^. To fully explore the continuous dynamic variations during the growing season, this study applied harmonic analysis to fit the NDVI and LSWI time series^45^. This method decomposes the original temporal signals into a set of harmonic coefficients with clear physical significance to represent crop phenological processes. By modeling the time series with periodic functions, this approach effectively suppresses high-frequency fluctuations caused by cloud cover, observational noise, and missing imagery, while enhancing the depiction of seasonal variation characteristics^46,47^ Through the integrated representation of NDVI and LSWI harmonic coefficients, different crop types exhibit distinct biophenological curves throughout their growth cycles, thereby increasing the inter-class separability in the feature space. These harmonic features not only retain the essential temporal information of crop growth but also improve robustness against inter-annual fluctuations, providing highly discriminative input features for subsequent RF classification.

1 $${NDVI}=\frac{{B}_{{NIR}}-{B}_{{Red}}}{{B}_{{NIR}}+{B}_{{Red}}}$$

2 $${LSWI}=\frac{\,{B}_{{NIR}}-{B}_{{SWIR}}}{{B}_{{NIR}}+{B}_{{SWIR}}}$$

**Fig. 4:**
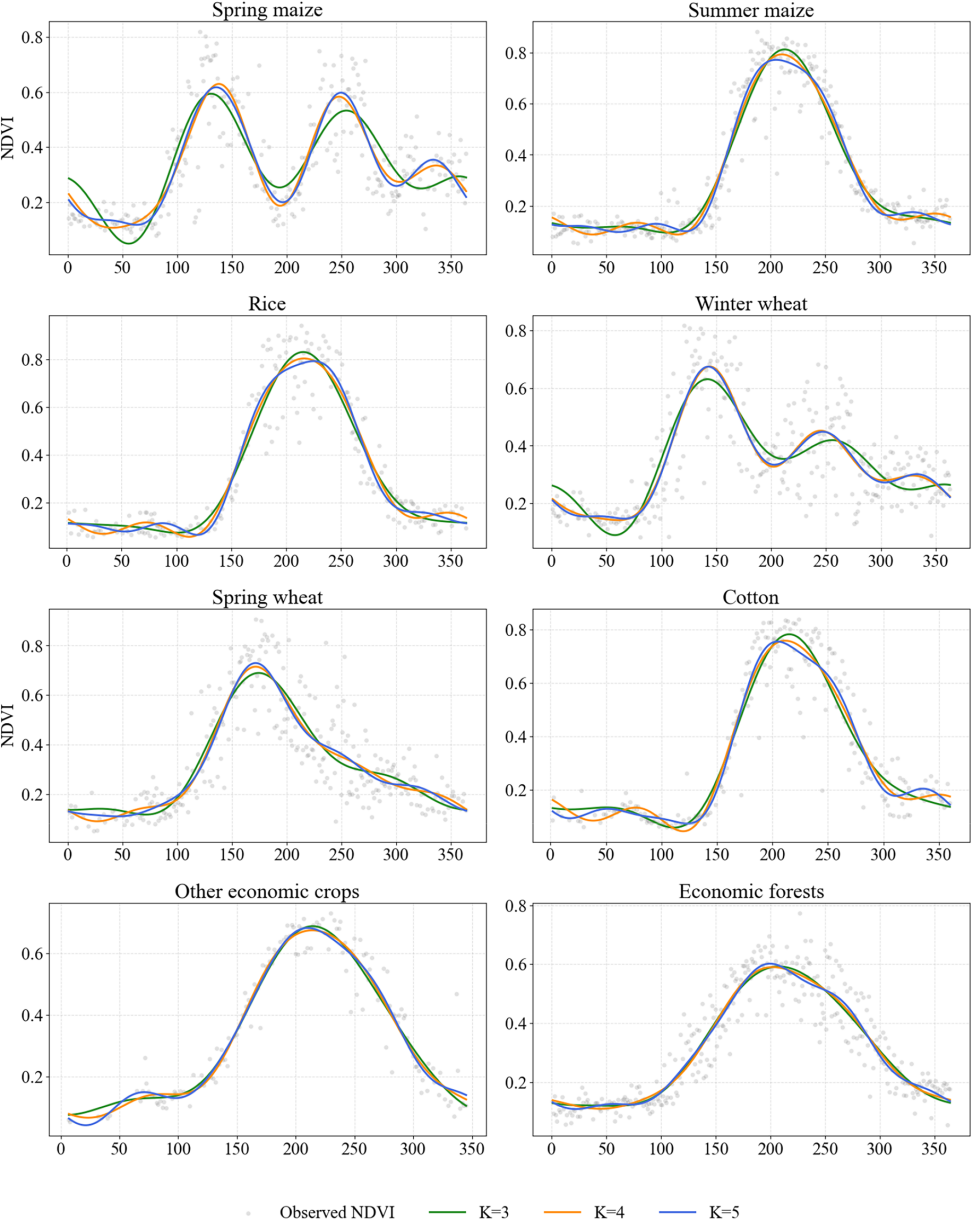
NDVI temporal profiles of major crop types fitted using harmonic functions.

Figure 4 illustrates the NDVI temporal characteristics of different crops fitted by harmonic functions. Overall, the biophenological curves of various crop types exhibit distinct differences in growing season timing, peak occurrence, and curve morphology. Winter wheat displays a characteristic curve with two peak growth periods, whereas spring wheat has a more concentrated growth cycle with an earlier peak. Annual crops such as summer maize, rice, and cotton show a typical unimodal structure, with NDVI reaching its maximum during the summer. In contrast, EF maintain a relatively stable NDVI throughout the year with minimal fluctuations.

At the level of harmonic features, the first-order harmonic primarily reflects the dominant cycle of intra-annual NDVI variation. Its amplitude and phase effectively characterize the differences in peak growth timing among crop types, providing strong discriminative power for broad crop category differentiation. Higher-order harmonics further capture the detailed morphological changes of the NDVI curves, describing the complexity of different crop time series. It should be noted that in this study, these higher-order harmonic features serve primarily as empirical temporal discriminators to enhance the separability of crop types in the feature space, rather than as direct indicators for interpreting specific physiological or agronomic processes. The differences in growth cycle structures and curve morphologies of NDVI time series across various crops provide stable and highly discriminative input features for machine-learning-based crop classification.

### Classification Strategy

During the classification phase, the RF algorithm was employed to identify crop types based on the extracted harmonic features. RF is an ensemble learning method that constructs multiple decision trees and aggregates their predictions to reduce model variance and enhance generalization performance. Compared with other machine learning algorithms, RF offers several notable advantages: it efficiently handles high-dimensional datasets without the need for dimensionality reduction, employs random sampling of both samples and features to minimize overfitting, exhibits strong robustness to noise, and provides stable and accurate predictions^48^. Owing to these strengths, RF has been widely applied in crop mapping, including studies on cotton classification in Xinjiang, China^6^, intra-seasonal mapping of winter wheat in China^49^, dynamic maize mapping in the United States^50^, and high-resolution mapping of single-season rice in China^51^.

In this study, the RF algorithm was employed to generate annual crop distribution maps for Xinjiang at a 30 m resolution from 2013 to 2024, leveraging the consistent multi-source observations provided by the HLSL30 product. We applied a cropland mask based on the CLCD to exclude non-cropland interference within the GEE platform. Ground sample points were then uploaded to GEE and used to train the built-in RF classifier. Two key parameters were optimized: *numberOfTrees* and *minLeafPopulation*. The *numberOfTrees* parameter determines the number of binary CART trees in the ensemble—more trees generally improve model stability but increase computational cost. To balance accuracy and efficiency, we incrementally increased *numberOfTrees* from 0 to 100 in steps of 5 and selected the model configuration yielding the highest Kappa coefficient as optimal. The *minLeafPopulation* parameter, which controls tree depth and helps prevent overfitting^52^, was fixed at 10. All other parameters were maintained at their default settings within the GEE RF classifier.

### Accuracy assessment

To comprehensively assess the accuracy of the crop classification results, this study adopted a combined quantitative evaluation and macro-level comparison strategy. First, an independent validation dataset—comprising samples not involved in model training—was used to construct confusion matrices. From these, key accuracy metrics were calculated, including Producer’s Accuracy (PA), User’s Accuracy (UA), and Overall Accuracy (OA), which together describe the classification performance across different crop categories. Detailed definitions and computational formulations of these metrics can be found in Foody (2021)^53^. In addition to sample-based validation, a regional-scale consistency assessment was conducted to evaluate the spatial reliability and statistical robustness of the generated crop maps. Specifically, crop area estimates derived from the HLSL30-based classification results were compared with officially reported planting areas for corresponding years, as published in the *Statistical Yearbook of China*. This comparative analysis provided an independent cross-check of the spatial accuracy and representativeness of the remote sensing classification results, ensuring that the derived crop distribution datasets are both statistically credible and regionally consistent.

## Data Records

**Fig. 5:**
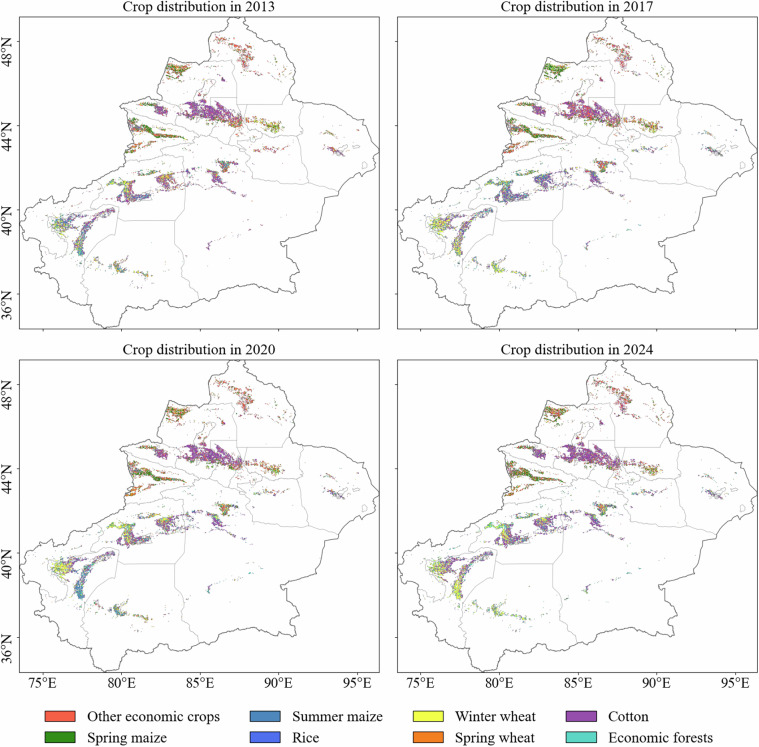
Spatial distribution of major crop types in Xinjiang. Panels (**a–d**) display the 30 m resolution crop maps for 2013, 2017, 2020, and 2024, respectively, generated from the HLSL30 dataset.

We generated a 30 m resolution map of major crops in Xinjiang for the period 2013–2024. This dataset provides a comprehensive characterization of the spatial distribution patterns of major crop types across the region (Fig. 5). The Xinjiang major crop distribution dataset is available at figshare^54^. The data are released in GeoTIFF format with a WGS84 (EPSG:4326) spatial reference.

## Technical Validation

### Accuracy verification

**Fig. 6:**
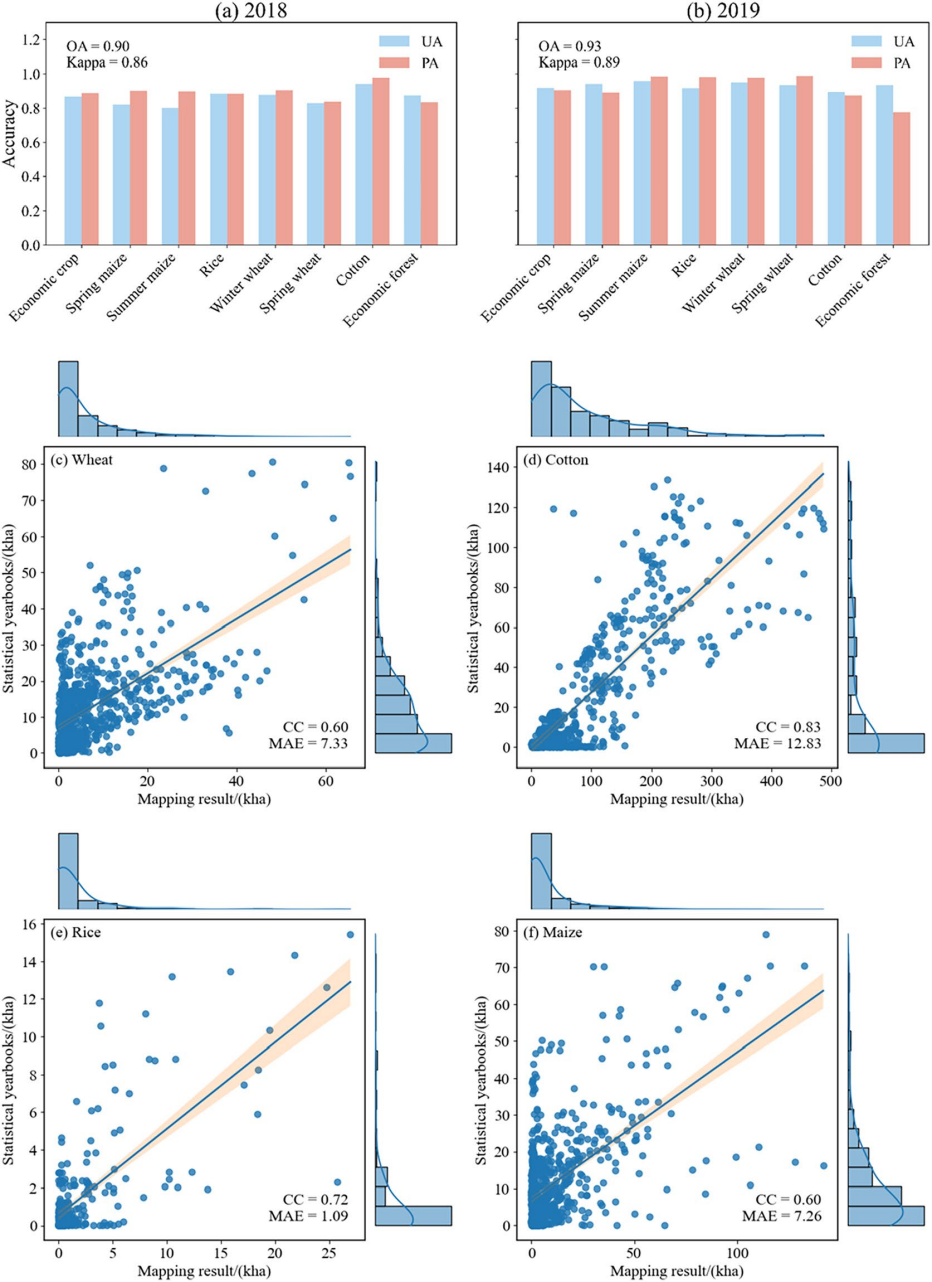
Validation of remote sensing–derived crop classification and area estimates using statistical yearbook data. (**a,b**) UA, PA, OA, and Kappa coefficient for 2018 and 2019, respectively. (**c**–**f**) Scatter plots showing the relationship between remotely sensed and county-level statistical crop area data for winter wheat, cotton, rice, and maize, respectively.

Figure 6 presents the accuracy assessment of the crop classification results for 2018 and 2019, along with a comparison between the estimated areas of major crops and official statistical yearbook data. Overall, the results for both years demonstrate high consistency and stability.

In terms of classification accuracy (Fig. 6a–b), the OA for 2018 was 0.90 with a Kappa coefficient of 0.86. The accuracy further improved in 2019, with an OA of 0.93 and a Kappa coefficient of 0.89, indicating strong overall reliability of the classification. The UA and PA for different crop types remained high, with most values exceeding 0.85. Major crops such as cotton, rice, and wheat exhibited stable and superior accuracy in both years. In contrast, the PA for EF was relatively lower, reflecting their high spatial heterogeneity and spectral overlap with other vegetation types.

Regarding area consistency verification (Fig. 6c–f), a comparative analysis between the remote-sensing-derived crop areas and county-level statistical data revealed a significant positive correlation for all four major crops. Cotton showed high correlation coefficients (CC = 0.83), indicating that remote sensing estimates effectively capture their spatial distribution and inter-annual variations. Although the correlation for wheat and maize was relatively lower (CC = 0.60), the overall trend remained consistent. The MAE varied across different crops; for instance, rice exhibited a higher MAE, which may be attributed to its smaller planting areas, fragmented spatial distribution, and discrepancies in statistical scales.

In summary, the results of both the accuracy assessment and area consistency verification demonstrate that the crop distribution data generated in this study are reliable at both the pixel and statistical scales. This dataset effectively characterizes the spatial patterns and area dynamics of major crops in Xinjiang, meeting the requirements for regional-scale agricultural monitoring and crop distribution analysis.

### Comparison with the existing map

**Fig. 7:**
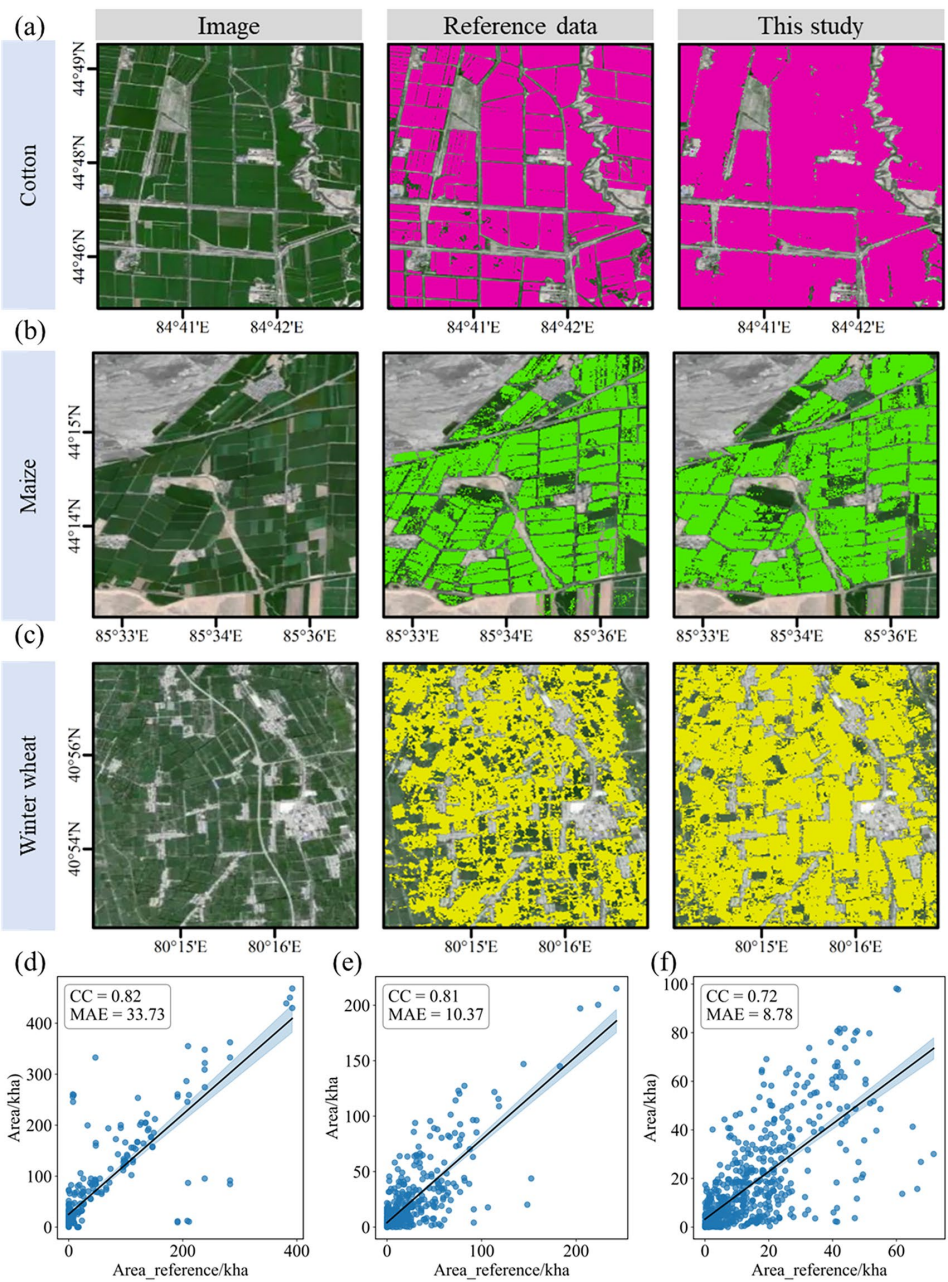
Validation of the produced crop distribution maps through spatial and statistical cross-comparisons with external datasets. Panels (**a**–**c**) show the spatial consistency of cotton, maize, and winter wheat, respectively, compared with external reference products. Panels (**d**–**f**) present the scatter plots and correlation analysis of the estimated crop areas against reference data for overlapping periods, aggregated at the county level using administrative boundaries.

Figure 7 illustrates the consistency between our crop mapping results and existing reference datasets at both local spatial and statistical scales. Specifically, Fig. 7(a–c) present a spatial comparison of remote sensing imagery, reference data, and our mapping results for cotton, maize, and winter wheat within typical agricultural zones. Overall, our crop distribution maps effectively reproduce the spatial patterns at the parcel scale, with crop boundaries, shapes, and continuity maintaining high consistency with the reference data. Although minor scattered discrepancies exist in localized areas, they are primarily distributed along field edges or in regions with high landscape complexity. These differences may stem from variations in mapping scales, temporal matching errors, and classification system definitions among different data sources.

Regarding area consistency verification (Fig. 7d–f), we cropped our mapping results and the reference data to the county level for overlapping years to perform a correlation analysis of the estimated crop areas. The results indicate a significant positive correlation between the remote-sensing-estimated areas and the reference data for all three major crops. Cotton achieved the highest correlation (CC = 0.82), followed by maize (CC = 0.81), while winter wheat was relatively lower (CC = 0.72). The MAE varied among the crops; cotton exhibited a relatively higher MAE, whereas maize and winter wheat showed lower error levels. This reflects the influence of planting scale, spatial continuity, and statistical uncertainties on the consistency assessment of crop area.

### Uncertainty analysis

Although the 30 m crop distribution dataset generated in this study exhibits high accuracy, several inherent uncertainties in long-term regional-scale mapping warrant discussion.

First, the temporal transferability of the classification model is a primary source of uncertainty. The model was trained on samples from 2018–2019, implicitly assuming that crop phenology and management practices remain stable over the 12-year period. However, factors such as climate variability, irrigation expansion, and shifts in agricultural policies in Xinjiang may induce phenological drifts or changes in cropping intensities^55,56^. While harmonic analysis is robust for fitting general trajectories, its focus on periodic features makes it less sensitive to abrupt shifts caused by extreme weather (e.g., droughts, floods)^17,46^. Consequently, uncertainties may increase in earlier (2013–2015) or anomalous years where actual phenological curves deviate from the typical patterns established during the sampling period.

Second, the internal heterogeneity of specific categories and the static nature of the cropland baseline pose classification challenges. Broad categories such as “Other Economic Crops (OEC)” and “Economic Forests (EF)” encompass diverse species with substantial spectral and phenological variability. We defined these as functional land-use types rather than species-level classes to balance regional-scale feasibility with thematic detail; however, this design inherently leads to lower accuracy for these minority classes due to spectral mixing and fragmented distributions^48,57^. Furthermore, relying on the CLCD mask may not fully capture annual boundary dynamics, such as the conversion of cropland to orchards or construction land, potentially introducing errors in transitional zones^23,58^.

Third, there is an unavoidable disparity between pixel-based remote sensing estimates and administrative statistical data. While our results show high macro-scale consistency with statistical yearbooks, the spatial implicit nature of statistics and differences in spatial scales (pixel vs. administrative unit) can affect area comparisons^7,59^. Moreover, validation intensity was uneven; extensive high-quality reference data for cotton and maize allowed for rigorous verification, whereas the evaluation of rice and OEC was constrained by the scarcity of external reference products. Thus, the validation performance of specific crops should not be indiscriminately generalized across all categories.

Looking forward, incorporating multi-source data (e.g., SAR, meteorological data) and integrating opportunistic citizen science data, such as geotagged street-view imagery^60,61^, could significantly alleviate the dependence on labor-intensive surveys and improve the model’s sensitivity to agroecological changes^9,19^.

## Data Availability

The dataset described in this study is publicly available in figshare at 10.6084/m9.figshare.30509831.

## Code availability

The GEE codes used to generate the cropland and cotton maps are available to the public at https://figshare.com/articles/dataset/_b_The_Multiscale_Mapping_Dataset_of_Major_Crops_in_Xinjiang_2013-2024_b_/30509831.

## References

[1] Pandey, P. C. & Pandey, M. Highlighting the role of agriculture and geospatial technology in food security and sustainable development goals. *Sustain. Dev.* **31**, 3175–3195 (2023).

[2] Kang, X. *et al*. The 10-m cotton maps in Xinjiang, China during 2018–2021. *Sci. Data* **10**, 688 (2023). 37816768 10.1038/s41597-023-02584-3 PMC10564865

[3] Johnson, D. M. Using the Landsat archive to map crop cover history across the United States. *Remote Sens. Environ.* **232**, 111286 (2019).

[4] Dong, J. *et al*. Early-season mapping of winter wheat in China based on Landsat and Sentinel images. (2020).

[5] Ni, R. *et al*. An enhanced pixel-based phenological feature for accurate paddy rice mapping with Sentinel-2 imagery in Google Earth Engine. *ISPRS J. Photogramm. Remote Sens.* **178**, 282–296 (2021).

[6] Tan, Z., Tan, Z., Luo, J. & Duan, H. Mapping 30-m cotton areas based on an automatic sample selection and machine learning method using Landsat and MODIS images. *Geo-Spat. Inf. Sci*. 1–18 10.1080/10095020.2023.2275622 (2023).

[7] Peng, Q. *et al*. A twenty-year dataset of high-resolution maize distribution in China. *Sci. Data* **10**, 658 (2023). 37752131 10.1038/s41597-023-02573-6 PMC10522722

[8] Boryan, C., Yang, Z., Mueller, R. & Craig, M. Monitoring US agriculture: the US Department of Agriculture, National Agricultural Statistics Service, Cropland Data Layer Program. *Geocarto Int.* **26**, 341–358 (2011).

[9] d’Andrimont, R. *et al*. From parcel to continental scale – A first European crop type map based on Sentinel-1 and LUCAS Copernicus *in-situ* observations. *Remote Sens. Environ.* **266**, 112708 (2021).

[10] Xuan, F. *et al*. Mapping crop type in Northeast China during 2013–2021 using automatic sampling and tile-based image classification. *Int. J. Appl. Earth Obs. Geoinformation* **117**, 103178 (2023).

[11] Hu, T., Hu, Y., Dong, J., Qiu, S. & Peng, J. Integrating Sentinel-1/2 Data and Machine Learning to Map Cotton Fields in Northern Xinjiang, China. *Remote Sens.* **13**, 4819 (2021).

[12] Han, L. *et al*. Monitoring Oasis Cotton Fields Expansion in Arid Zones Using the Google Earth Engine: A Case Study in the Ogan-Kucha River Oasis, Xinjiang, China. *Remote Sens.* **14**, 225 (2022).

[13] Han, X. *et al*. Spatiotemporal changes in agricultural planting structure in the Turpan–Hami Basin, Xinjiang, China: Remote sensing monitoring from 1990 to 2023. *Smart Agric. Technol.* **12**, 101626 (2025).

[14] Hao, P., Wang, L. & Niu, Z. Comparison of Hybrid Classifiers for Crop Classification Using Normalized Difference Vegetation Index Time Series: A Case Study for Major Crops in North Xinjiang, China. *PLOS ONE* **10**, e0137748 (2015). 26360597 10.1371/journal.pone.0137748 PMC4567051

[15] Li, X. *et al*. Mapping annual 10-m maize cropland changes in China during 2017–2021. *Sci. Data* **10**, 765 (2023). 37925513 10.1038/s41597-023-02665-3 PMC10625519

[16] Qiu, B. *et al*. Maps of cropping patterns in China during 2015–2021. *Sci. Data* **9**, 479 (2022). 35931696 10.1038/s41597-022-01589-8 PMC9356131

[17] Yao, J. *et al*. Recent climate and hydrological changes in a mountain–basin system in Xinjiang, China. *Earth-Sci. Rev.* **226**, 103957 (2022).

[18] Yang, G. *et al*. Numerical assessment of the effect of water-saving irrigation on the water cycle at the Manas River Basin oasis. *China. Sci. Total Environ.* **707**, 135587 (2020). 31784147 10.1016/j.scitotenv.2019.135587

[19] Yang, L. *et al*. Google Earth Engine and Artificial Intelligence (AI): A Comprehensive Review. *Remote Sens.* **14**, 3253 (2022).

[20] Zhang, C. *et al*. Towards automation of in-season crop type mapping using spatiotemporal crop information and remote sensing data. *Agric. Syst.* **201**, 103462 (2022).

[21] Feng, L. *et al*. Review of the technology for high-yielding and efficient cotton cultivation in the northwest inland cotton-growing region of China. *Field Crops Res.* **208**, 18–26 (2017).

[22] Yu, J. *et al*. Evaluating sustainable intensification levels of dryland agriculture: A focus on Xinjiang. *China. Ecol. Indic.* **158**, 111448 (2024).

[23] Yang, J. & Huang, X. The 30 m annual land cover dataset and its dynamics in China from 1990 to 2019. *Earth Syst. Sci. Data* **13**, 3907–3925 (2021).

[24] Yang, J. & Huang, X. The 30 m annual land cover datasets and its dynamics in China from 1985 to 2025. *Zenodo*10.5281/ZENODO.18180184 (2026).

[25] Nasa, J. P. L. NASADEM Merged DEM Global 1 arc second V001. *NASA Land Processes Distributed Active Archive Center*10.5067/MEASURES/NASADEM/NASADEM_HGT.001 (2020).

[26] Neigh, C. *et al*. HLS Operational Land Imager Surface Reflectance and TOA Brightness Daily Global 30m v2.0. *NASA Land Processes Distributed Active Archive Center*10.5067/HLS/HLSL30.002 (2021).

[27] Ju, J. *et al*. The Harmonized Landsat and Sentinel-2 version 2.0 surface reflectance dataset. *Remote Sens. Environ.* **324**, 114723 (2025).

[28] Claverie, M. *et al*. The Harmonized Landsat and Sentinel-2 surface reflectance data set. *Remote Sens. Environ.* **219**, 145–161 (2018).

[29] Bolton, D. K. *et al*. Continental-scale land surface phenology from harmonized Landsat 8 and Sentinel-2 imagery. *Remote Sens. Environ.* **240**, 111685 (2020).

[30] Chen, N., Tsendbazar, N.-E., Hamunyela, E., Verbesselt, J. & Herold, M. Sub-annual tropical forest disturbance monitoring using harmonized Landsat and Sentinel-2 data. *Int. J. Appl. Earth Obs. Geoinformation* **102**, 102386 (2021).

[31] Zhou, Q. *et al*. Monitoring Landscape Dynamics in Central U.S. Grasslands with Harmonized Landsat-8 and Sentinel-2 Time Series Data. *Remote Sens.* **11**, 328 (2019).

[32] Deng, Y. *et al*. Detecting the onset of rice field inundation in the Lower Mississippi River Basin via Harmonized Landsat Sentinel-2 (HLS) satellite time series. *ISPRS J. Photogramm. Remote Sens.* **228**, 28–43 (2025).

[33] Hong, C. *et al*. The role of harmonized Landsat Sentinel-2 (HLS) products to reveal multiple trajectories and determinants of cropland abandonment in subtropical mountainous areas. *J. Environ. Manage.* **336**, 117621 (2023). 36870318 10.1016/j.jenvman.2023.117621

[34] Hao, P., Tang, H., Chen, Z., Yu, L. & Wu, M. High resolution crop intensity mapping using harmonized Landsat-8 and Sentinel-2 data. *J. Integr. Agric.* **18**, 2883–2897 (2019).

[35] Liu, X. *et al*. Comparisons between temporal statistical metrics, time series stacks and phenological features derived from NASA Harmonized Landsat Sentinel-2 data for crop type mapping. *Comput. Electron. Agric.* **211**, 108015 (2023).

[36] Dong, J. *et al*. Early-season mapping of winter wheat in China based on Landsat and Sentinel images. *Earth Syst. Sci. Data* **12**, 3081–3095 (2020).

[37] Dong, J. *et al*. Annual winter wheat mapping dataset in China from 2001 to 2020. *Sci. Data* **11**, 1218 (2024). 39532896 10.1038/s41597-024-04065-7 PMC11557595

[38] Fu, Y. *et al*. High-resolution mapping of global winter-triticeae crops using a sample-free identification method. *Earth Syst. Sci. Data* **17**, 95–115 (2025).

[39] Shen, R. *et al*. A 30 m Resolution Distribution Map of Maize for China Based on Landsat and Sentinel Images. *J. Remote Sens.* **2022**, 2022/9846712 (2022).

[40] Weigand, M., Staab, J., Wurm, M. & Taubenböck, H. Spatial and semantic effects of LUCAS samples on fully automated land use/land cover classification in high-resolution Sentinel-2 data. *Int. J. Appl. Earth Obs. Geoinformation* **88**, 102065 (2020).

[41] You, N. & Dong, J. Examining earliest identifiable timing of crops using all available Sentinel 1/2 imagery and Google Earth Engine. *ISPRS J. Photogramm. Remote Sens.* **161**, 109–123 (2020).

[42] Zhang, X. *et al*. Monitoring vegetation phenology using MODIS. *Remote Sens. Environ.* **84**, 471–475 (2003).

[43] Bao, G. *et al*. Modeling net primary productivity of terrestrial ecosystems in the semi-arid climate of the Mongolian Plateau using LSWI-based CASA ecosystem model. *Int. J. Appl. Earth Obs. Geoinformation* **46**, 84–93 (2016).

[44] Xiao, X. *et al*. Mapping paddy rice agriculture in southern China using multi-temporal MODIS images. *Remote Sens. Environ.* **95**, 480–492 (2005).

[45] Kalair, A., Abas, N., Kalair, A. R., Saleem, Z. & Khan, N. Review of harmonic analysis, modeling and mitigation techniques. *Renew. Sustain. Energy Rev.* **78**, 1152–1187 (2017).

[46] Dong, L. *et al*. Shifting agricultural land use and its unintended water consumption in the North China Plain. *Sci. Bull.* **69**, 3968–3977 (2024). 10.1016/j.scib.2024.11.009 39550272

[47] Sakamoto, T. *et al*. Detecting temporal changes in the extent of annual flooding within the Cambodia and the Vietnamese Mekong Delta from MODIS time-series imagery. *Remote Sens. Environ.* **109**, 295–313 (2007).

[48] Li, H., Zhang, C., Zhang, S. & Atkinson, P. M. Crop classification from full-year fully-polarimetric L-band UAVSAR time-series using the Random Forest algorithm. *Int. J. Appl. Earth Obs. Geoinformation* **87**, 102032 (2020).

[49] Yang, G. *et al*. Automated in-season mapping of winter wheat in China with training data generation and model transfer. *ISPRS J. Photogramm. Remote Sens.* **202**, 422–438 (2023).

[50] Wen, Y. *et al*. Mapping corn dynamics using limited but representative samples with adaptive strategies. *ISPRS J. Photogramm. Remote Sens.* **190**, 252–266 (2022).

[51] Shen, R. *et al*. High-resolution distribution maps of single-season rice in China from 2017 to 2022. *Earth Syst. Sci. Data* **15**, 3203–3222 (2023).

[52] Pelletier, C., Valero, S., Inglada, J., Champion, N. & Dedieu, G. Assessing the robustness of Random Forests to map land cover with high resolution satellite image time series over large areas. *Remote Sens. Environ.* **187**, 156–168 (2016).

[53] Foody, G. M. Impacts of ignorance on the accuracy of image classification and thematic mapping. *Remote Sens. Environ.* **259**, 112367 (2021).

[54] Liang, Q. A 30 m Multi-Year Dataset of Major Crop Distributions in Xinjiang, China. *Based on Harmonized Landsat–Sentinel-2 Data*. 1243707220 Bytes figshare 10.6084/M9.FIGSHARE.30509831 (2013–2024) (2026). 10.1038/s41597-026-07082-w PMC13172342 41872228

[55] Wu, B. *et al*. Challenges and opportunities in remote sensing-based crop monitoring: a review. *Natl. Sci. Rev.* **10**, nwac290 (2023). 36960224 10.1093/nsr/nwac290 PMC10029851

[56] Wang, Y., Feng, L., Zhang, Z. & Tian, F. An unsupervised domain adaptation deep learning method for spatial and temporal transferable crop type mapping using Sentinel-2 imagery. *ISPRS J. Photogramm. Remote Sens.* **199**, 102–117 (2023).

[57] Wang, S., Azzari, G. & Lobell, D. B. Crop type mapping without field-level labels: Random forest transfer and unsupervised clustering techniques. *Remote Sens. Environ.* **222**, 303–317 (2019).

[58] Xin, Q. *et al*. Satellite mapping of maize cropland in one-season planting areas of China. *Sci. Data* **10**, 437 (2023). 37419886 10.1038/s41597-023-02334-5 PMC10328911

[59] Hu, Q. *et al*. Integrating coarse-resolution images and agricultural statistics to generate sub-pixel crop type maps and reconciled area estimates. *Remote Sens. Environ.* **258**, 112365 (2021).

[60] Zhong, L., Hu, L. & Zhou, H. Deep learning based multi-temporal crop classification. *Remote Sens. Environ.* **221**, 430–443 (2019).

[61] Yan, Y. & Ryu, Y. Exploring Google Street View with deep learning for crop type mapping. *ISPRS J. Photogramm. Remote Sens.* **171**, 278–296 (2021).

